# Hedgehog overexpression leads to the formation of prostate cancer stem cells with metastatic property irrespective of androgen receptor expression in the mouse model

**DOI:** 10.1186/1423-0127-18-6

**Published:** 2011-01-18

**Authors:** Han-Hsin Chang, Bo-Yie Chen, Chia-Yung Wu, Zih-Jay Tsao, Ying-Yu Chen, Chin-Pao Chang, Chi-Rei Yang, David Pei-Cheng Lin

**Affiliations:** 1 School of Nutrition, Chung Shan Medical University, Taichung 402, Taiwan; 2 School of Optometry, Chung Shan Medical University, Taichung 402, Taiwan; 3 Division of Urology, Changhua Christian Hospital, Changhua 500, Taiwan; 4 Division of Urology, Taichung Veterans General Hospital, Taichung 407, Taiwan; 5 School of Medical Laboratory and Biotechnology, Chung Shan Medical University, Taichung 402, Taiwan; 6 Department of Urology, Chung Shan Medical University Hospital, Taichung 402, Taiwan

## Abstract

**Background:**

Hedgehog signalling has been implicated in prostate tumorigenesis in human subjects and mouse models, but its effects on transforming normal basal/stem cells toward malignant cancer stem cells remain poorly understood.

**Methods:**

We produced pCX-shh-IG mice that overexpress Hedgehog protein persistently in adult prostates, allowing for elucidation of the mechanism during prostate cancer initiation and progression. Various markers were used to characterize and confirm the transformation of normal prostate basal/stem cells into malignant cancer stem cells under the influence of Hedgehog overexpression.

**Results:**

The pCX-shh-IG mice developed prostatic intraepithelial neoplasia (PIN) that led to invasive and metastatic prostate cancers within 90 days. The prostate cancer was initiated through activation of P63^+ ^basal/stem cells along with simultaneous activation of Hedgehog signalling members, suggesting that P63^+^/Patch1^+ ^and P63^+^/Smo^+ ^cells may serve as cancer-initiating cells and progress into malignant prostate cancer stem cells (PCSCs). In the hyperplastic lesions and tumors, the progeny of PCSCs differentiated into cells of basal-intermediate and intermediate-luminal characteristics, whereas rare ChgA^+ ^neuroendocrine differentiation was seen. Furthermore, in the metastatic loci within lymph nodes, kidneys, and lungs, the P63^+ ^PCSCs formed prostate-like glandular structures, characteristic of the primitive structures during early prostate development. Besides, androgen receptor (AR) expression was detected heterogeneously during tumor progression. The existence of P63^+^/AR^-^, CK14^+^/AR^- ^and CD44^+^/AR^- ^progeny indicates direct procurement of AR^- ^malignant cancer trait.

**Conclusions:**

These data support a cancer stem cell scenario in which Hedgehog signalling plays important roles in transforming normal prostate basal/stem cells into PCSCs and in the progression of PCSCs into metastatic tumor cells.

## Background

Adult prostate epithelial stem cells reside within the basal cell layer and possess high self-renewal capacity, leading to the generation of intermediate, luminal, and neuroendocrine cell lineages [1,2]. Normally, most of the adult prostate epithelial stem cells differentiate into intermediate cells without requirement of androgen receptor (AR) activity. The process is characterized by loss of P63 and gain of CK14 or CD44 expression [3-5]. Then, the intermediate cells undergo terminal differentiation as they form the luminal cells with CK8 expression and become dependent on AR activity for maintenance and fulfilling of their functions [3,4].

Like many other cancer stem cells, a hypothesis of prostate cancer stem cells (PCSCs) originated from normal stem cells has been proposed based on their highly tumorigenic trait and basal/stem cell-like properties of self-renew and differentiation [6-9]. The hypothesis has been supported by some recent studies indicating that androgen-refractory prostate cancer cells contain an apparent basal/stem cell-like signature [9-12], suggesting that these cancer cells may not be derived from the AR^+ ^luminal cell population [9]. In other words, AR signalling may be entirely bypassed during the transformation of prostate stem cells into PCSCs [9]. Alternatively, AR signalling may remain active at early stages of transformation but become repressed as the cancer cells eventually progress into an AR-independent status [13]. The entire bypass pathway has attracted much attention recently, since the basal/stem cells in human prostates are AR^(- or low) ^[14], likely to be the direct origin of androgen-independent cancer cells through tumorigenic transformation, although there has been no evidence so far to support this hypothesis.

Hedgehog (Hh) signalling plays a key role in stem cell plasticity and in many developmental, physiological, and pathogenetic processes [15]. Binding of the Hedgehog ligand to the Patched 1 (Patch1) receptor releases the Patch1-associated Smoothened (Smo) G-protein, which triggers a cascade of intracellular signalling activations that lead to the binding of downstream transcription factors, e.g., Gli1, Gli2 and Gli3 to their target sequences and then expression of target genes involved in the control of cell division or differentiation [16]. Aberrant Hh signalling activation has been implicated in prostate tumorigenesis in human subjects and mouse models [17-22]. Previously, we had confirmed that Hh signalling members are expressed in tumorigenic P63^+ ^basal cells in human specimens and these cells are capable of differentiation into multiple lineages, suggesting that Hh signalling may promote primary prostatic cancer stem cells [20]. However, the tumorigenic activation of basal/stem cells and their progression toward a metastatic status under the influence of Hh signalling remain to be further elucidated. To further characterize the basal cells during tumorigenic activation, we established a mouse prostate cancer model in which prostate tumorigenesis was induced from a normal status through persistent Hh overexpression [21], taking advantage of using mouse models to elucidate tumor formation and evaluate candidate therapeutic agents [23,24].

In this study, we used the Hh overexpression mouse model to elucidate whether the PCSCs arise from P63^+ ^basal/stem cells and to examine whether these cancer cells can maintain stem cell characteristics after metastasis. More importantly, we intended to elucidate whether P63^+ ^basal/stem cells can be directly transformed into AR^- ^cancer cells. We demonstrated that Hh overexpression initiated malignant transformation of P63^+ ^basal/stem cells that subsequently differentiated into both AR^+ ^and AR^- ^progeny of the basal-intermediate and intermediate-luminal progeny, but rare ChgA^+ ^neuroendocrine cells. The Hh-initiated P63^+ ^basal/stem cells were characteristic of PCSCs, as they were able to form primitive prostate-like glandular structures in the metastatic loci. Besides, androgen receptor (AR) expression was detected heterogeneously in PCSCs when they were differentiated into intermediate and luminal cells, indicating that androgen were not necessary for these PCSCs (AR^-^). These data challenge the model of AR^+ ^transition into androgen-independent PCSCs and suggest a potentially better treatment strategy by inhibition of Hedgehog signalling prior to androgen-deprivation therapy.

## Methods

### Plasmid vectors

Mouse Shh-expressing pCX-shh-IG vector and pCX-IG vehicle control vectors were kindly provided by Dr. Kerby C. Oberg, Loma Linda University [25]. The pCX-shh-IG vector contains a Shh insert tagged with green fluorescence protein (GFP) sequence driven by a CMV promoter. The vehicle control pCX-IG vector contains the same CMV promoter and the GFP tag, but without the Shh insert. Therefore, presence of GFP in prostates indicated successful introduction of pCX-shh-IG vector and expression of Hh protein.

### Intraprostatic injection and electroporation

ICR strain male mice aged 8-10 weeks were purchased from National Laboratory Animal Center, Academia Sinica, Taipei for use in this study. The mice were anesthetized and exposed of their prostate glands by surgery, followed by intraprostatic injection and electroporation to introduce the pCX-shh-IG or pCX-IG vectors as described in our previous study [21]. All animal procedures were performed following the Guide for the Care and Use of Laboratory Animal that had been promulgated by the Institute of Laboratory Animal Resources and had been approved by the animal care and use committee of Chung Shan Medical University.

### Immunohistochemical staining, double-immunofluorescence staining and TUNEL assay

Standard procedures were followed to prepare prostate tissue sections for immunohistochemistry. Antigen retrieval was achieved by boiling tissue in citrate buffer (pH 6.0) for 20 min. Primary antibodies (all at 1:50 dilution) were goat anti-Shh antibody (N-19), rabbit anti-Patch1 (H-267), goat anti-Patch1 (G-19), rabbit anti-Smo (H-300), rabbit anti-Gli1 (H-300), goat anti-Gli2 (N-20), goat anti-Gli3 (N-19), goat anti-CK14 (C-14), and mouse anti-CD44 (DF-1485); all were purchased from Santa Cruz Biotechnology (Santa Cruz, CA). The mouse anti-p63 (4A4), mouse anti-CK8 (TS1), rabbit anti-AR (RB-9030), mouse anti-PCNA (MS-106), rabbit anti-ChgA (RB-9003) and mouse anti-tubulin (MS-581) were purchased from Lab Vision Corporation (Lab Vision, Fremont, CA). The secondary antibodies were horseradish peroxidase- conjugated anti-mouse, anti-goat, and anti-rabbit IgG (all at 1:200) purchased from Jackson ImmunoResearch Laboratories, Inc., PA. For double-immunofluorescence detection, primary antibodies were applied simultaneously, followed by incubation with donkey anti-mouse rhodamine Red-X and FITC, anti-rabbit rhodamine Red-X and FITC anti-goat FITC (1:50) (Jackson ImmunoResearch Laboratories, Inc., PA) and counterstained with DAPI. Standard brightfield and immunofluorescence microscopy were performed for photography using a Zeiss Axioskop2 Plus microscope and SoftWoRx software. In situ apoptosis assay (TUNEL assay) was processed following the manufacturer's instruction (Millipore, Billerica, MA).

### Western blot analysis

Standard procedures were performed for western blot analysis. Briefly, protein extract (150 μg) was fractionated on SDS-polyacrylamide electrophoresis gel and transferred to a polyvinylidine difluoride membrane (Immobilon-P, Millipore). The membrane was then incubated with primary antibody (described above) overnight at 4°C, followed by incubation with secondary antibody (horseradish peroxidase-conjugated anti-mouse, anti-rabbit, or anti-goat IgG) for 1 hour. The immune complexes on membranes were detected by chemiluminescence methods (ECL, Amersham).

### Quantification of positive or double-positive cells

Tissue sections from five prostates of the pCX-IG-injected vehicle controls and five pCX-shh-IG-injected mouse prostates were used. Each prostate specimen was from an individual mouse. In each specimen, three randomly picked 1000 μm^2 ^boxes showing the normal, PIN or the CaP sites were used for quantification. The results were presented as the average percentage of double-positive cells over total counted cells. The difference between the normal, PIN, and the CaP sites was analyzed by Student's t test (significant when p < 0.001).

## Results

### Persistent Hedgehog overexpression induced mouse prostate tumorigenesis

**Figure 1:**
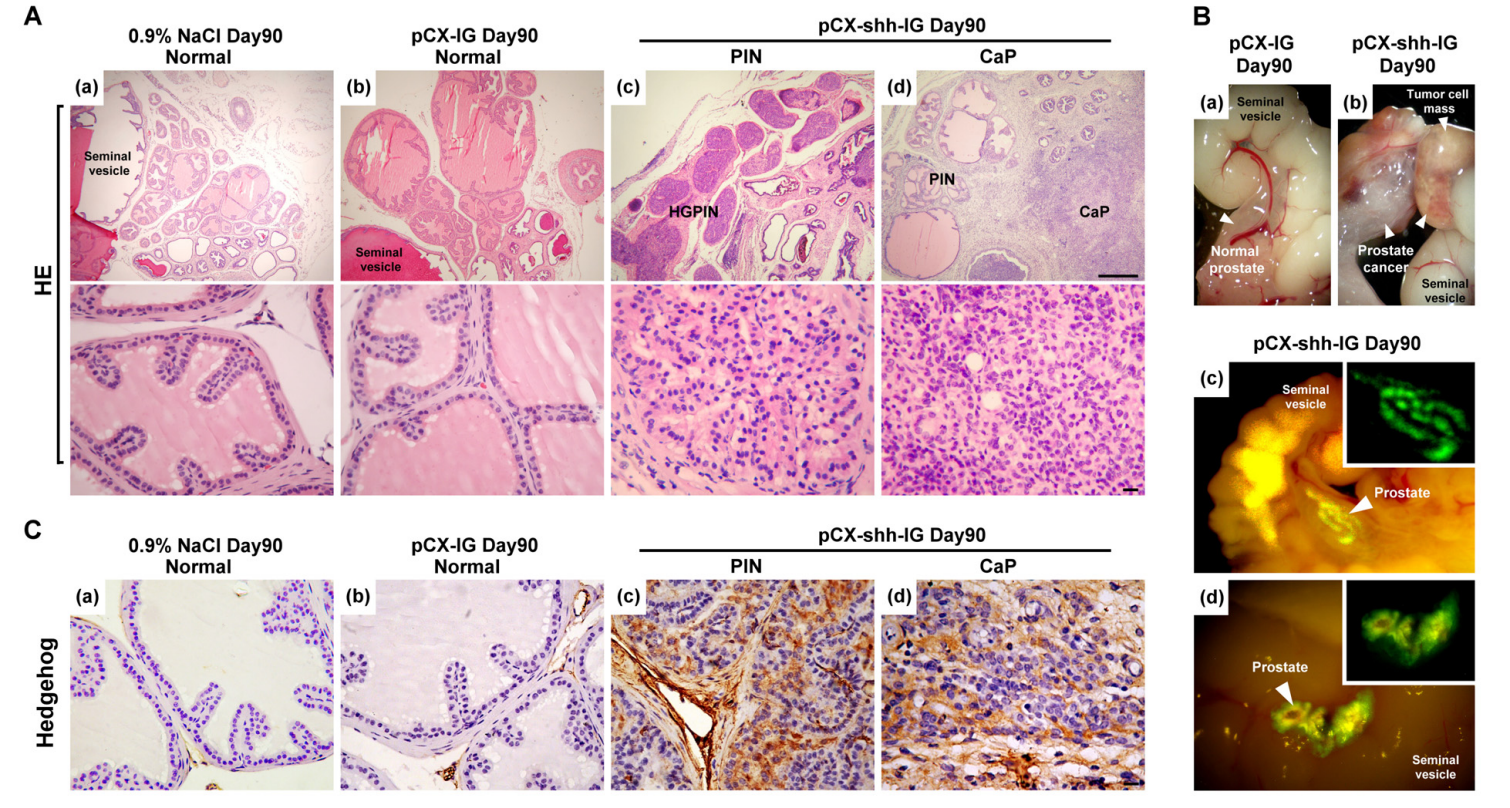
**Persistent Hedgehog overexpression induces mouse prostate tumorigenesis**. (A) Histopathological analysis with hematoxylin-eosin stain showing characteristics of progressive tumor formation through stages of PIN and CaP in a pCX-shh-IG-injected prostate at day 90 as compared to sham (0.9%NaCl) and vehicle (pCX-IG) injection controls. The lower pictures are higher magnifications corresponding to the tissue sections shown in the upper pictures. (B) Tumor formation induced by Hedgehog overexpression (arrowheads in (b) indicate tumor mass), as compared to vehicle control (arrowhead in (a)). The prostate gland displayed GFP signals at day 90 after pCX-shh-IG injection (arrowhead-indicated in (c) and (d), also magnified in the inlets). (C) The pCX-shh-IG-injected prostate tissue sections were stained strongly for Hedgehog protein in PIN and CaP, in contrast to those of the sham and vehicle controls. Scale bars: 50 μm in upper (d) of panel A; 10 μm in lower (d) of panel A and in (d) of panel C. CaP: prostate cancer; HE: hematoxylin-eosin stain; PIN: prostatic intraepithelial neoplasia; HGPIN: high grade prostatic intraepithelial neoplasia.

**Figure 2:**
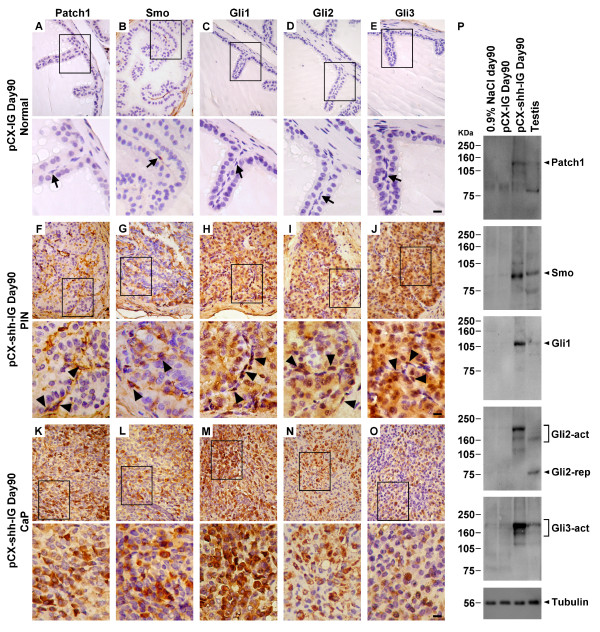
**Overexpression of Hedgehog signaling members, including Patch1, Smo, Gli1, Gli2, and Gli3, in pCX-shh-IG-injected prostates at day 90 after injection**. The lower pictures are magnifications of the boxed areas shown in the upper pictures. Hedgehog signalling members were expressed in the PIN (F-J) and CaP (K-O) of the pCX-shh-IG-injected prostates, in contrast to the absence of evident signal in the pCX-IG-injected vehicle controls (A-E; basal/stem cells indicated by arrows). Note that some cells positive for Hedgehog signalling (indicated by arrowheads in F-J) appeared to be basal/stem cells in morphology. Western blot analysis (P) confirmed that Hedgehog signalling members were highly expressed in the pCX-shh-IG- injected prostates, as compared to those sham injections with 0.9% saline or vehicle controls with pCX-IG vector. Tubulin detections served as loading controls. Scale bars: 10 μm in lower E, lower J and lower O. CaP: prostate cancer; PIN: prostatic intraepithelial neoplasia; Gli2-act: active form of Gli2; Gli2-rep: repressed form of Gli2; Gli3-act: active form of Gli3.

The pCX-shh-IG-injected mouse prostates exhibited discernible tumors on day 90 after the preparation, which was not observed in those of the pCX-IG-injected vehicle controls and the sham injection controls (with 0.9% NaCl). The tumors, found exclusively in the prostates, showed characteristics of progressive tumorigenesis through stages of prostatic gland hyperplasia, prostatic intraepithelial neoplasia (PIN), and prostate cancer (CaP) (Figure 1A). The pCX-shh-IG-injected prostates exhibited strong Hh protein expression in contrast to the vehicle controls (Figure 1C). Evident Patch1, Smo, Gli1, Gli2, and Gli3 expressions were found in PIN lesions (Figure 2F to 2J) and CaP (Figure 2K to 2O) of the pCX-shh-IG-injected prostates, in contrast to the almost absence of expression in the vehicle controls (Figure 2A to 2E), except that few basal cells and stromal cells were Smo^+ ^(Figure 2B). The Patch1 and Smo staining patterns were consistent with their membrane localizations (Figure 2F to 2G and 2K to 2L), while Gli transcription factors were predominantly detected in the nucleus and cytoplasm (Figure 2H to 2J and 2M to 2O). Moreover, Hh signaling proteins were expressed heterogeneously, i.e. only in some cell lineages of the PIN lesions (Figure 2F to 2J; indicated by arrowheads) and particularly evident in the round-shaped or accumulated basal cells (indicated by arrowheads in the magnified areas of Figure 2F to 2J), in contrast to the normal slim and flat basal cells (indicated by arrows in the magnified areas of Figure 2A to 2E). These data strongly suggest that the prostate cancer cells are likely to be transformed from quiescent basal cells under the influence of Hh overexpression. The data were further solidified by immunoblotting assay showing similar results (Figure 2P). Patch1, Smo, Gli1, Gli2 and Gli3 proteins were found highly expressed in the pCX-shh-IG-injected prostates even on day 90 after the preparation, in contrast to the absence or minimal expression in the vehicle controls or sham controls (Figure 2P). Activated forms of Gli2 and Gli3 proteins were dominantly detected in the pCX-shh-IG-injected prostates (Figure 2P; indicated by Gli2-act and Gli3-act), but not in the vehicle and sham controls. Thus, this preparation offers a suitable mouse model to study the effects of persistent Hh overexpression during prostate cancer initiation and progression.

### P63^+ ^basal/stem cells were activated by Hedgehog overexpression

**Figure 3:**
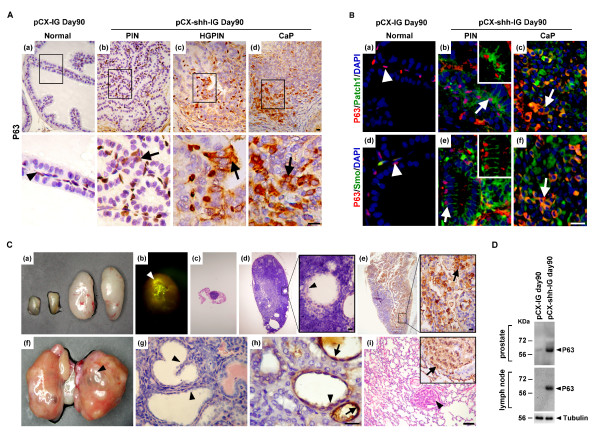
**P63^+ ^basal cells are involved in prostate tumorigenesis and progressed into metastatic cancer cells with Hedgehog overexpression**. The boxed areas in (A) and (C) are magnified respectively and inlets in (B) correspond to arrow-indicated areas. (A) Increased P63^+ ^basal/stem cell density and altered cellular morphology, including bigger cell size, disoriented polarity, and displaced localization were found along with tumor initiation and progression in the pCX-shh-IG-injected prostate (arrow-indicated in (b), (c) and (d)), in contrast to the normal P63^+ ^cells in the pCX-IG-injected prostate (indicated by arrowhead in (a)). (B) Patch1 and Smo proteins were located within P63^+ ^basal cells in the PIN and CaP of pCX-shh-IG-injected prostate. The P63^+^/Patch1^+ ^and P63^+^/Smo^+ ^cells are arrow-indicated respectively in (b) and (e), as compared to those in the pCX-IG-injected prostate (arrowhead in (a) and (d)). Patch1 or Smo was co-expressed with P63^+ ^in some cancer cells of the advanced prostate cancer (arrow-indicated respectively in (c) and (f)). (C) P63^+ ^cancer cells recapitulated prostate-like glandular structure formation in the metastatic loci (arrowhead-indicated in (d), (f), (g), (h) and (i)). Lymph node metastasis is shown by two enlarged specimens on the right of (a) and in (b), (d), (e), as compared to the normal small lymph node in (c) and the two other small specimens on the left side of (a). Note that GFP signals can be detected in the lymph node in (b). Kidney metastasis is shown in (f-h) and lung metastasis in (i). Arrows in (e), (h), and (i) indicate P63^+ ^metastatic cancer cells. (D) Western blot analysis confirmed increased P63^+ ^cells in the prostates and the lymph nodes of the pCX-shh-IG-injected mice. All scale bars: 10 μm. CaP: prostate cancer; PIN: prostatic intraepithelial neoplasia; HGPIN: high grade prostatic intraepithelial neoplasia.

To understand whether P63^+ ^basal/stem cells were activated under the influence of Hedgehog overexpression, we examined the pCX-shh-IG-injected prostates during the progression of PIN toward CaP to gain further insights (Figure 3A). The P63^+ ^basal/stem cells in the pCX-shh-IG-injected prostates showed characteristic features of activation, including increased cell density, bigger cell size, disoriented polarity, and displaced localization (Figure 3A; arrow-indicated in (b)). These features are comparable to those observed in BCH (basal cell hyperplasia) of human prostate specimens [25], in contrast to the few P63^+ ^cells lying flat along the basement membrane of the vehicle controls (Figure 3A; arrowhead-indicated in (a)). Moreover, apart from nucleus localization in the normal (Figure 3A; arrowhead-indicated in (a)) and hyperplasic basal cells (Figure 3A; arrow-indicated in (b)), P63 was detected in the cytoplasm of cells in the HGPIN (high grade PIN) lesions (Figure 3A; arrow-indicated in (c)) and CaP (Figure 3A; arrow-indicated in (d)). Interestingly, P63 was expressed in some but not all populations of prostate cancer cells (Figure 3A; (d)), similar to that observed in human prostate cancers [20].

### Hedgehog overexpression promoted P63^+ ^basal/stem cell hyperplasia toward malignant transformation

Since prostate tumorigenesis was induced and P63^+ ^basal/stem cells exhibited human BCH-like features of cellular activation in the pCX-shh-IG-injected prostates [20], it is tempting to examine whether Patch1 receptor and its co-receptor Smo are activated in the P63^+ ^basal/stem cells. Patch1 and Smo were found highly expressed in the P63^+ ^basal/stem cells of the pCX-shh-IG-injected prostates, being located in the cell membrane of P63^+ ^basal cells in regions of primary PIN lesions (Figure 3B; arrow in (b) indicated P63^+^/Patch1^+ ^and arrow in (e) indicated P63^+^/Smo^+ ^cells). In contrast, only very limited Patch1 or Smo expression was detected in the quiescent P63^+ ^basal cells in the vehicle controls (Figure 3B; arrowhead-indicated in (a) and (d)). In advanced CaP lesions, P63^+ ^cancer cells were seen persistently co-expressed with Patch1 or Smo protein and contributed to a certain portion of the heterogeneous cancer cell populations (Figure 3B; arrow in (c) indicated P63^+^/Patch1^+ ^and arrow in (f) indicated P63^+^/Smo^+ ^cells). These data support Hedgehog involvement in promotion of basal cell hyperplasia toward malignant transformation.

### Metastatic P63^+ ^cancer cells recapitulated prostate-like primitive glandular structure formation in the metastatic loci

To understand whether the P63^+ ^cancer cells induced by Hedgehog overexpression were characteristic of PCSCs, we examined their stemness property and capacity of metastasis. Since pCX-shh-IG vector was only introduced in the prostates, the GFP signals were expected to be detected only within the prostates (Figure 1B; (c) and (d)). Ectopic GFP signals in other organs would indicate prostate cancer cell metastasis (Figure 3C; indicated by arrowhead in (b)). Based on the presence of GFP signals, we found metastasis in the mouse lymph nodes (19/27), kidneys (7/27), and lungs (5/27) following 90 days after introducing pCX-shh-IG vector, but not in the vehicle and sham controls (Figure 3C; (a) indicated two small lymph nodes (on the left) from the sham controls and two enlarged lymph nodes (on the right) from the pCX-shh-IG-injected mice). The metastatic loci of lymph nodes (Figure 3C; (a), (b), (d) and (e)), kidneys (Figure 3C; (f), (g) and (h)), and lungs (Figure 3C; (i)) were infiltrated with P63^+ ^cells (Figure 3C; indicated by arrows in (e), (h) and (i)). The increase of P63^+ ^cells in the pCX-shh-IG-injected prostates and lymph nodes after metastasis was confirmed by western blot analysis at day 90 after the preparation (Figure 3D). Furthermore, the P63^+ ^cells within most of the metastatic loci displayed a prostate-like primitive glandular structure (Figure 3C; indicated by arrowheads in (d), (f), (g), (h) and (i)). This finding demonstrated recapitulation of prostate formation by the transformed P63^+ ^basal/stem cells in the metastatic loci. The data evidently demonstrated both cancer cell and stem cell characteristics of the transformed P63^+ ^basal/stem cells, supporting that they could be the origin of PCSCs under the influence of persistent Hedgehog overexpression.

### P63^+ ^basal/stem cells were transformed into AR^+ ^or AR^- ^cancer cells but rarely into ChgA^+ ^neuroendocrine cancer cells

**Figure 4:**
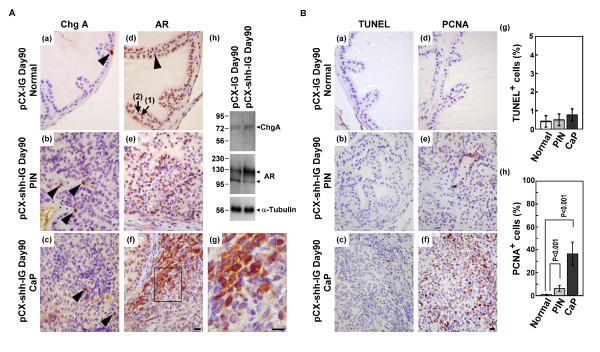
**Characterization of mouse prostate cancer under the influence of Hedgehog overexpression**. (A) The ChgA^+ ^neuroendocrine cells were rarely detected in the pCX-IG-injected vehicle control prostate (arrowhead-indicated in (a)) and in PIN and CaP lesions of the pCX-shh-IG-injected prostates (arrowhead-indicated in (b) and (c)). All luminal cells (arrowhead-indicated in (d)) and some basal cells (arrow (1)-indicated in (d)) in the normal prostate expressed AR in the nucleus, in contrast to the nucleus or cytosolic AR localization in the PIN and CaP lesions ((e), (f) and (g)). Some AR^- ^basal cells (arrow (2)-indicated in (d)) and AR^- ^progeny of PCSCs were found in the CaP ((f) and the magnified boxed area of (f) as shown in (g)). Western blot analysis confirmed the status of ChgA and AR expression in the vehicle control and pCX-shh-IG-injected prostate ((h)). (B) The PIN and CaP lesions ((b), (c) and (g)) showed similar apoptosis status as compared to the normal prostate ((a) and (g)). PCNA^+ ^proliferative cells were increased in the PIN and CaP lesions ((e), (f) and (h)) as compared to normal prostate ((d) and (h)). All scale bars represent 10 μm in length. CaP: prostate cancer; PIN: prostatic intraepithelial neoplasia; HGPIN: high grade prostatic intraepithelial neoplasia.

Conventional human prostate cancer cells include malignant cells of luminal, basal or neuroendocrine origin at various proportions and unlike the ChgA^- ^luminal cancer cells, tumor cells of neuroendocrine origin are ChgA^+ ^[26]. We found only few ChgA^+ ^neuroendocrine cells in the PIN lesions and CaP of the pCX-shh-IG-injected prostates (Figure 4A; (b) and (c); indicated by arrowheads), not much different to what was found in the vehicle controls (Figure 4A; (a); indicated by arrowhead). In the pCX-shh-IG-injected prostates, both the AR^+ ^and AR^- ^cells were detected, with the AR protein, if present, located in the nucleus or cytosol in the cells of PIN lesions and CaP (Figure 4A; (e-g)). Wereas, in the vehicle controls, AR was predominantly located in the nucleus of luminal cells (Figure 4A; (d); indicated by arrowhead) and of some basal cells (Figure 4A; (d); arrow (1) indicated AR^+ ^basal cells and arrow (2) indicated AR^- ^basal cells). This finding was confirmed by western blot analysis at day 90 after the injection (Figure 4A; (h)). Since cytosolic or no AR protein was observed in some of the cancer cells, it appeared that androgens were not necessarily required for survival of these cells or otherwise they should had been programmed into cell death. To elucidate the situation, we performed TUNEL assay and found no significant apoptosis in PIN lesions and CaP of pCX-shh-IG-injected prostates (Figure 4B; (b), (c) and (g)), as compared to normal vehicle controls (Figure 4B; (a) and (g)). In contrast, PCNA^+ ^proliferative cells were increased in the PIN and CaP lesions (Figure 4B; (e), (f) and (h)), but rare in the vehicle controls (Figure 4B; (d) and (h)). These observations indicated that the PCSCs, as induced by persistent Hedgehog overexpression, did not commit to differentiate into ChgA^+ ^neuroendocrine cells and might bypass AR signalling for survival and proliferation.

### PCSCs progeny differentiated into basal-intermediate and intermediate-luminal cells

**Figure 5:**
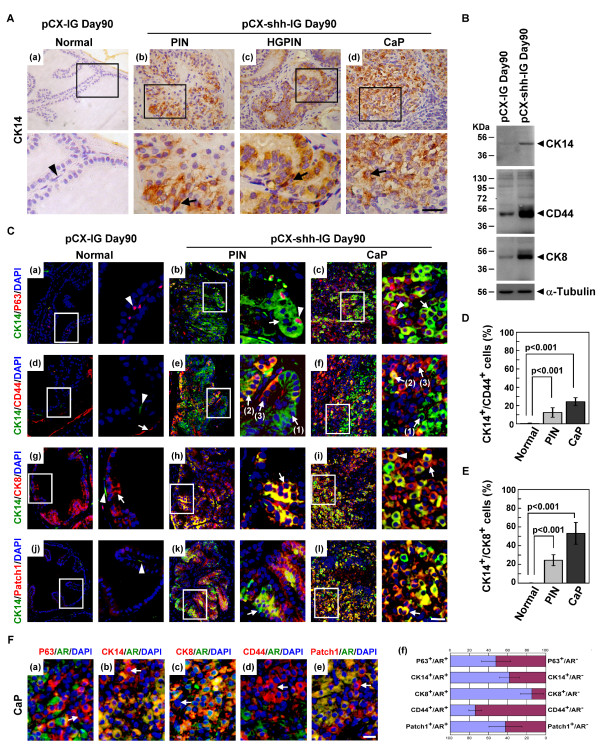
**Differentiation status and AR expression profile of PCSCs under the influence of Hedgehog overexpression**. The boxed areas in the pictures are further magnified and shown in the corresponding lower or right pictures. (A) Increased CK14^+ ^cells along with tumorigenic progression in the pCX-shh-IG-injected prostate ((b), (c) and (d)), as compared to the normal prostate ((a)). (B) Western blot analysis indicated up-regulation of CK14, CD44 and CK8 in the pCX-shh-IG-injected prostates as compared to the normal pCX-IG-injected prostates. (C) Characterization of PCSCs by double-immunofluorescence staining showing differentiation toward CK14^+ ^progeny ((a), (b) and (c)), CK14^+ ^cells toward CD44^+ ^progeny ((d), (e) and (f)), and CK14^+ ^cells toward CK8^+ ^progeny ((g), (h) and (i)). CK14^+ ^differentiation involved Hedgehog signalling activation, as indicated by co-localized Patch1 expression ((j), (k) and (l)). The basal-intermediate (CK14^+^/CD44^+^) and intermediate-luminal (CK14^+^/CK8^+^) populations were increased in PIN and CaP of pCX-shh-IG-injected prostates as compared to those of the pCX-IG-injected vehicle controls (D and E). (F) Some PCSCs were AR^- ^as indicated by arrows in (a), (b), (c), (d), and (e), even though they were P63^+^, CK14^+^, CK8^+^, CD44^+^, or Patch1^+^. The relative proportions of AR^+ ^and AR^- ^cells among P63^+^, CK14^+^, CK8^+^, CD44^+^, and Patch1^+ ^cell populations were shown respectively in (f). All scale bars represent 10 μm in length. CaP: prostate cancer; PIN: prostatic intraepithelial neoplasia; HGPIN: high grade prostatic intraepithelial neoplasia.

Since our data had indicated that ChgA^+ ^neuroendocrine cells did not constitute the main cellular lineage under the influence of Hh overexpression, the differentiation status of PCSCs was examined with some cell markers, including P63 (primitive basal cells), CK14 (advanced and hyperplastic basal cells), CD44 (intermediate cells), and CK8 (mature luminal cells). In the normal vehicle control prostates, fewer CK14^+ ^basal cells lay flat along the basement membrane (Figure 5A; arrowhead-indicated in (a)), whereas in the PIN and CaP lesions of pCX-shh-IG-injected prostates, CK14^+ ^cells were increased with neoplastic transformation (Figure 5A; arrow-indicated in (b), (c) and (d)). By western blot analysis, we found that CK14, CD44, and CK8 markers were up-regulated in the prostate tumors as compared to the normal prostates (Figure 5B). Double labelling of P63 and CK14 markers showed P63^+^/CK14^(low or -) ^cells in the normal prostates (Figure 5C; arrowhead-indicated in (a)). In the pCX-shh-IG-injected prostates, as the prostates were induced into PIN and progressed into CaP status, these P63^+^/CK14^(low or -) ^cells appeared to be differentiated into P63^+^/CK14^+ ^(Figure 5C; arrowhead-indicated in (b)) and further into P63 ^(low)^/CK14^+ ^(Figure 5C; arrowhead-indicated in (c)) and P63^-^/CK14^+ ^(Figure 5C; arrow-indicated in (b) and (c)) cells. The loss of P63 expression revealed that the PCSCs were differentiated toward the luminal progeny. Comparably, the CK14^+^/CD44^+ ^cancer cells in the PIN and CaP lesions (Figure 5C; arrow (2)-indicated in (e) and (f)) were likely to be originated from CK14^(low or -)^/CD44^(low or -) ^cells in the normal prostates (Figure 5C; arrowhead-indicated in (d)) or from CK14^+^/CD44^(low or -) ^cells (Figure 5C; arrow (1)-indicated in (e) and (f)) and CK14^(low or -)^/CD44^+ ^cells (Figure 5C; arrow (3)-indicated in (e) and (f)), as the cells were transformed into PIN and progressed into CaP conditions. Additionally, the CK14^+^/CK8^+ ^cancer cells in the PIN and CaP lesions (Figure 5C; arrow-indicated in (h) and (i)) might be originated from CK14^(low or -)^/CK8^- ^cells in the normal prostates (Figure 5C; arrowhead-indicated in (g)), and then differentiated into CK14^low^/CK8^(high or ^^+) ^progeny (Figure 5C; arrowhead-indicated in (i)). The increase of basal-intermediate (CK14^+^/CD44^+^) (Figure 5D) and intermediate-luminal (CK14^+^/CK8^+^) (Figure 5E) progeny in the PIN and CaP lesions of pCX-shh-IG-injected prostates were significant when compared to the vehicle controls. The involvement of Hh signalling during PCSCs differentiation in the PIN and CaP lesions was indicated by double labelling of Patch1 and CK14 (Figure 5C; arrow-indicated in (k) and (l)), in contrast to the only few Patch1^+ ^cells detected in the normal vehicle controls (Figure 5C; arrowhead-indicated in (j)).

### The PCSCs progeny differentiation was not totally dependent on androgen-AR axis

To examine whether malignant differentiation of PCSCs might be independent from the androgen-AR axis in the pCX-shh-IG-injected prostates, we co-localized AR with different markers, including P63, CK14, CK8, CD44, and Patch1 (Figure 5F). Apart from the AR^+ ^cells, we detected P63^+^/AR^-^, CK14^+^/AR^-^, CD44^+^/AR^- ^and even the CK8^+^/AR^- ^cells in the CaP (Figure 5F). The concurrent existence of AR^+ ^and AR^- ^cell populations indicated that androgen-AR axis was not indispensably required for PCSCs and their progeny to undergo further differentiation (Figure 5F; (f)). The localization of Patch1 protein in both AR^+ ^and AR^- ^cancer cells (Figure 5F; (e)) revealed the involvement of Hedgehog signalling in both cell lineages.

## Discussion

Basal/stem cells are the origin of all prostate intra-glandular cells [1,27] and P63 has been proposed to be required for prostate stem cell plasticity and differentiation [27,28]. Thus, PCSCs are potentially originated from normal P63^+ ^prostate stem cells, although there has been no evidence so far to support this hypothesis. In our previous study analyzing the human specimen [20], we demonstrated that Hh protein is expressed in P63^+ ^basal cells, in correlation with basal cell hyperplasia and CaP formation. Our previous data support normal prostate stem cells transformation into PCSCs, although based only on retrospective observations.

In this study, we provide the evidence to further support PCSCs derivation from normal prostate stem cells, based on several lines of observations. Firstly, the entire process of prostate tumorigenesis was reconstituted in vivo and the effects of Hh overexpression on the normal prostate stem cells was shown by the transformation of P63^+^/Patch1^(Low or -) ^and P63^+^/Smo ^(Low or -) ^quiescent basal cells into the P63^+^/Patch1^+ ^and P63^+^/Smo^+ ^hyperplastic basal cells, comparable to the human BCH condition that had been previously observed [20]. Secondly, the P63^+ ^cells showed major cancer cell and stem cell peculiarity on the metastatic sites. The P63^+ ^cells were not only present within various metastatic loci, but also differentiated into prostate-like glandular structures where they were located within the basal compartment. Thirdly, the P63^+ ^basal/stem cells, after being transformed into malignant cells, were capable of differentiation into the basal-intermediate (P63^+^/CK14^+^) and intermediate-luminal (CK14^+^/CD44^+ ^and CK14^+^/CK8^+^) progeny, and rarely the ChgA^+ ^neuroendocrine lineage.

PCSCs derivation from normal prostate stem cells has also been supported by several lines of evidence. Purified cells such as Sca-1^+ ^and CD49f^+ ^cells from the mouse prostates [29,30] or CD44^+^, CD133^+^, and α2/β1 integrin^+ ^cells from the human prostates [31,32] were used in renal capsule transplantation studies. These normal cells could generate prostate-like structures in the kidney, supporting the presence of prostate progenitor/stem cells in these purified cell populations and their ability to regenerate in ectopic sites. Such ectopic regeneration capacity has been proven to retain even after the transformation of normal prostate stem cells into PCSCs. For example, AR^- ^P63^-^CD44^+^Nestin^+ ^HPET-5 cells were purified from prostate cancer cell lines [33] and shown to recapitulate prostate-like structures in the mouse kidney after transplantation. Besides, a previous report showed that Hedgehog may recapitulate embryonic gene expression in tumor myofibroblasts [34]. Despite the aforementioned studies, in vivo prostate cancer cell metastasis with a stem cell peculiarity to generate prostate-like structures in the metastatic loci has not been reported. In this study, we demonstrated the infiltration of P63^+ ^cancer cells in the metastatic loci and generation of prostate-like glandular structures. Our data support the prostate cancer stem cell characteristics observed in previous studies and, to our knowledge, these are the first data to confirm that PCSCs metastasis occurs under in vivo conditions.

Clinically, it is known that advanced metastatic androgen-independent prostate cancers exhibit more basal/stem cell-like differentiation although the underlying mechanism remains unclear [9,10]. The transformation of basal/stem cells into PCSCs was proposed as a possible mechanism. In such case, the PCSCs are supposed to maintain high proliferation and differentiation capacities without AR activity [35,36], since androgen is considered not necessary for the survival of basal/stem cells; in contrast to the terminally-differentiated luminal cells which require androgen and AR for survival [7,9,37]. Our data showed transition of nuclear to cytoplasmic P63 expression and such transition had been reported to associate with higher proliferative activity, reduced apoptosis, and increased mortality [35]. This may explain the abundant AR^- ^cancer cells found in many high grade prostate cancers [38-40] and the failure of androgen deprivation therapies. Alternatively, persistent AR activation due to loss-of-function mutations has been reported in some androgen-refractory prostate cancers [13,41]. Therefore, both AR^+ ^and AR^- ^cells were capable of forming androgen-independent prostate cancer cells as the tumorigenesis progresses to the more advanced stages. In this study, both the AR^+ ^and AR^- ^prostate cancer cells were generated under the effects of persistent Hedgehog signalling activation in the mouse model (Figure 4A and Figure 5F). Our data is consistent with the findings of Hedgehog signalling activation in several human prostate cancers [17-20,22], especially in the androgen-independent prostate cancers [22,42,43]. Particularly, we showed that some basal/stem cancer cells were AR^-^, e.g. P63^+^/AR^-^, CK14^+^/AR^- ^and CD44^+^/AR^- ^(Figure 5F; arrowhead-indicated in (a), (b) and (d)). These sub-populations of AR^- ^cancer cells were most likely to contribute directly to the androgen-independent tumors. In fact, androgen ablation by castration could not reduce tumor mass in this model (data not shown). Here, the key findings in this study have confirmed that overexpression of Hedgehog can transform prostate basal cells *in vivo *and lead the transformed cells to progress into aggressive AR^- ^PCSCs progeny. Although the nuclear AR^+ ^(active form) and cytoplasmic AR^+ ^(inactive form) cells were both observed in the aggressive tumors in this *in vivo *model and our data showed that Hedgehog signalling activation may substitute the androgen-AR axis for tumor survival or malignant transformation, the underlying mechanisms remain to be further investigated by using *in vitro *studies.

## Conclusions

Our data support the hypothesis that P63^+ ^hyperplastic basal cells targeted by Hh overexpression may be the true cellular origin of primary prostate cancer. This study also supports that inhibition of Hedgehog signalling may be a better treatment strategy for androgen-independent tumors prior to androgen-deprivation therapy.

## Competing interests

The authors declare that they have no competing interests.

## Authors' contributions

HHC, BYC, and DPL designed the study, carried out production of pCX-shh-IG and pCX-IG mice, and contributed to the writing of manuscript. CYW helped with animal maintenance, plasmid vector preparation, immunohistochemical and double-immunofluorescence staining. ZJT performed TUNEL assay and western blot analysis. CPC and CRY carried out the quantification of positive and double-positive cells.
